# Transplantation of Adipose-Tissue-Engineered Constructs with CRISPR-Mediated UCP1 Activation

**DOI:** 10.3390/ijms24043844

**Published:** 2023-02-14

**Authors:** Svetlana Michurina, Iurii Stafeev, Maria Boldyreva, Vu Anh Truong, Elizaveta Ratner, Mikhail Menshikov, Yu-Chen Hu, Yelena Parfyonova

**Affiliations:** 1 National Medical Research Centre of Cardiology Named after Academician E. I. Chazov, 121552 Moscow, Russia; 2 Faculty of Biology, Lomonosov Moscow State University, 119991 Moscow, Russia; 3 Cell and Molecular Biology Unit, Faculty of Biology and Biotechnology, National Research University Higher School of Economics, 101000 Moscow, Russia; 4 Department of Chemical Engineering, National Tsing Hua University, Hsinchu 300044, Taiwan; 5 Frontier Research Center on Fundamental and Applied Sciences of Matters, National Tsing Hua University, Hsinchu 300044, Taiwan; 6 Faculty of Basic Medicine, Lomonosov Moscow State University, 119991 Moscow, Russia

**Keywords:** CRISPR, tissue engineering, thermogenesis, adipocytes

## Abstract

Thermogenic adipocytes have potential utility for the development of approaches to treat type 2 diabetes and obesity-associated diseases. Although several reports have proved the positive effect of beige and brown adipocyte transplantation in obese mice, translation to human cell therapy needs improvement. Here, we describe the application of CRISPR activation (CRISPRa) technology for generating safe and efficient adipose-tissue-engineered constructs with enhanced mitochondrial uncoupling protein 1 (UCP1) expression. We designed the CRISPRa system for the activation of UCP1 gene expression. CRISPRa-UCP1 was delivered into mature adipocytes by a baculovirus vector. Modified adipocytes were transplanted in C57BL/6 mice, followed by analysis of grafts, inflammation and systemic glucose metabolism. Staining of grafts on day 8 after transplantation shows them to contain UCP1-positive adipocytes. Following transplantation, adipocytes remain in grafts and exhibit expression of PGC1α transcription factor and hormone sensitive lipase (HSL). Transplantation of CRISPRa-UCP1-modified adipocytes does not influence glucose metabolism or inflammation in recipient mice. We show the utility and safety of baculovirus vectors for CRISPRa-based thermogenic gene activation. Our findings suggest a means of improving existing cell therapy approaches using baculovirus vectors and CRISPRa for modification and transplantation of non-immunogenic adipocytes.

## 1. Introduction

Obesity is a state of impaired balance between energy consumption and expenditure, dramatically increasing the risk for developing type 2 diabetes mellitus (T2DM), cardiovascular diseases and cancer [1,2]. Adipose tissue, which consists mainly of adipocytes, is essential for whole-body energy homeostasis due to its unique lipid metabolism, as well as endocrine and thermogenic functions [3]. The increase in thermogenic adipose tissue has strong potential for preventing metabolic complications due to energy dissipation and combustion of energetic substrates [4,5,6,7]. 

Thermogenic brown and beige adipocytes express uncoupling protein 1 (UCP1), which dissipates H^+^ gradients on mitochondrial inner membranes, decreasing the efficiency of ATP synthesis and releasing energy from the oxidation of glucose and fatty acids in the form of heat. In addition to the canonical thermogenesis, UCP1-independent mechanisms also exist. They include recently discovered Ca^2+^, creatine and TAG futile cycling mechanisms [8]. However, UCP1-dependent thermogenesis is the most studied, and significant data on its application in models of obesity have been accumulated. The activation and constitutive expression of UCP1 in white adipose tissue reduces total body weight in obese mice and pigs [9,10,11]. Conversely, genetic ablation of UCP1 leads to obesity and hyperglycemia [12,13].

UCP1 expression can be increased in response to cold conditions and thermogenic activators such as β-adrenoceptors agonists [14]. Nevertheless, pharmacological stimulation of thermogenesis is still not widely adopted due to side effects of thermogenic activators, such as cardiovascular outcomes [15]. In this light, the application of gene and cell therapy with further transplantation of thermogenic cells is an alternative path to metabolic health [7,16]. Several reports have described the engineering and transplantation of brown-like adipocytes with upregulated UCP1 expression [17,18]. Although these reports demonstrated the positive effects of UCP1-expressing adipocytes on metabolism, technologies used for gene regulation have limitations. CRISPR gene editing has low efficiency and off-target effects, and lentiviral vectors integrate into the genome and alter gene transcription [19,20,21,22]. To improve the development of UCP1-expressing adipocytes for clinical translation, we suggest CRISPRa technology, delivered by baculovirus vectors. 

Transcriptional regulation based on CRISPR expanded the possibilities of genetic engineering in various fields including adipocyte biology [17,23]. CRISPR-based transcription activation systems use deactivated Cas9 (dCas9) and sgRNAa, which attract the complex of transcriptional activators p65 and heat shock factor 1 (HSF1) to the promoters of the target gene. The CRISPRa system has high efficiency and low off-target-effects [24,25]. The efficiency of modification can be increased by delivering all of the components of the CRISPRa-UCP1 system in one vector. The baculovirus expression system, with packaging capacity up to 38 kb, is advantageous in comparison to adeno-associated and lentiviral approaches with limited packaging capacity. Baculovirus is an insect-derived-vector that shows high transduction efficiency in various cell lines. Moreover, the lack of replication and integration in the genome in mammalian cells makes baculovirus a safe instrument for gene regulation and future clinical translation [26]. 

In this study, we describe a concept for the generation of UCP1-expressing adipocytes from adipose-derived stem cells (ADSCs) using a novel CRISPRa technology for endogenous gene regulation, delivered by a safe and efficient baculovirus system. Adipose tissue engineering constructs are vascularized, contain intact adipocytes with increased UCP1 expression after transplantation in mice and do not stimulate local and systemic inflammation. Adipose tissue engineering using baculovirus transduction of ADSC is a promising tool for the improvement of metabolic health and the prevention of complications. 

## 2. Results

### 2.1. CRISPRa System Efficiently Increases UCP1 Expression and Activates Thermogenesis in Adipocytes 

The development of CRISPRa-engineered adipose tissue constructs includes (1) isolation of subcutaneous ADSC from lean mice, (2) adipogenic differentiation of cells, (3) baculovirus transduction and (4) subcutaneous transplantation of adipocytes suspended in Matrigel. We regulated the expression of UCP1, the effector of canonical thermogenesis in brown and beige adipocytes. Previous studies have demonstrated the high potential of UCP1 activation to increase energy expenditure and improve metabolic health. We explore the usability of the CRISPRa system for UCP1 activation and apply a safe and efficient baculovirus delivery system for adipocyte modification. The design and development of the CRISPRa system was described in detail in a previous study [27]. Produced baculovirus particles contain bacmid DNA, encoding dCas9, an MPH transcription-activation complex of MCP-p65-HSF1 proteins and an array of four activating UCP1-targeting sgRNAas that had two MS2 hairpins for MPH recruitment. First, we analyzed the CRISPRa system efficiency with respect to UCP1 regulation and the impact of the modification on thermogenesis and lipid content in adipocytes in vitro.

**Figure 1:**
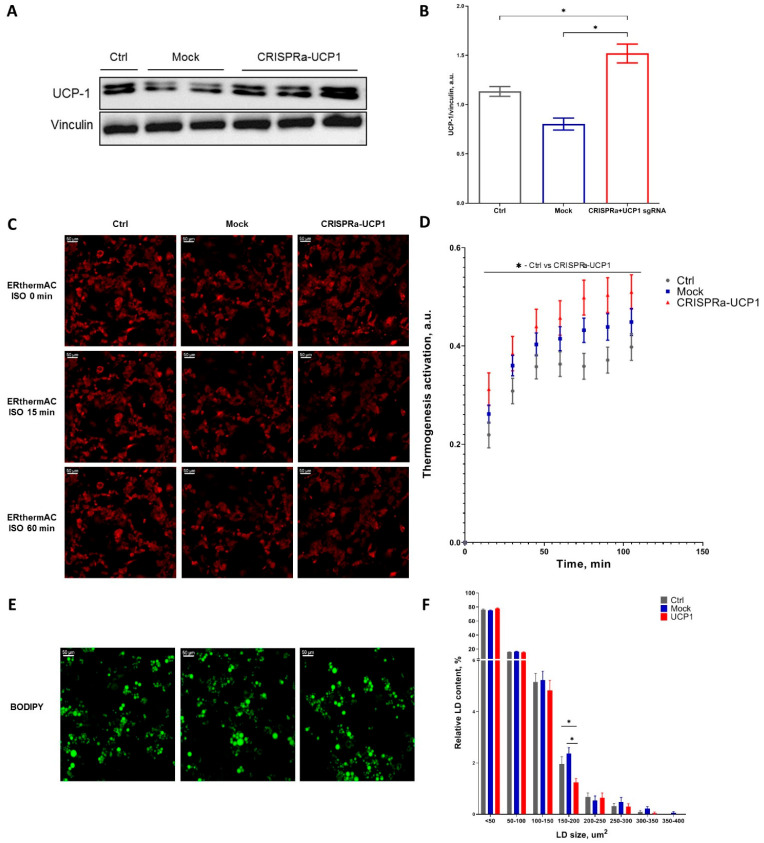
CRISPRa-UCP1 modification of mature adipocytes increased UCP-1 expression, leading to thermogenic activity and a decrease in medium-sized lipid droplet (LD) accumulation. (**A**) A representative Western blot membrane; (**B**) densitometry of Western blots; (**C**) representative images of adipocytes stained with ERthermAC; (**D**) time-dependent curve of thermogenesis activation under isoproterenol stimulation (ISO); (**E**) representative images of adipocytes stained with BODIPY493/503; (**F**) size distribution of LD. Data are presented as mean ± SEM, one way ANOVA test, *—significance threshold *p* < 0.05.

We confirmed that seven days after CRISPRa-UCP1 transduction, adipocytes had elevated UCP1 expression in comparison to control and mock groups (Figure 1A,B). The increased expression of UCP1 is able to activate thermogenic energy dissipation in adipocytes. Indeed, CRISPRa-UCP1-modified cells demonstrated increased thermogenesis activation in response to ISO, which is the canonical thermogenesis activator (Figure 1C,D). The difference between CRISPRa-UCP1 and control groups was significant, but the mock group also had non-significantly elevated thermogenesis activation. In connection with thermogenesis activation, we also analyzed LD accumulation in adipocytes, because it could be affected by lipolysis and oxidative metabolism stimulation during thermogenesis. We found that in CRISPRa-UCP1 adipocytes the number of medium-sized LDs (150–200 μm^2^) was significantly decreased. However, the number of small and large LDs was not changed. Thus, we confirmed UCP1 expression and thermogenesis activation in CRISPRa-UCP1 adipocytes, which can be further applied in vivo.

### 2.2. Transplanted Adipocytes Retain High UCP1 Expression

To confirm the suitability of the CRISPRa-UCP1 system for in vivo translation, we first produced adipose-tissue-engineered constructs by transplantation of the suspension of modified adipocytes with Matrigel matrix in mice. Eight days after transplantation of tissue-engineered constructs, we assessed the presence of UCP1-positive adipocytes in grafts and determined the CRISPRa system’s efficiency.

**Figure 2:**
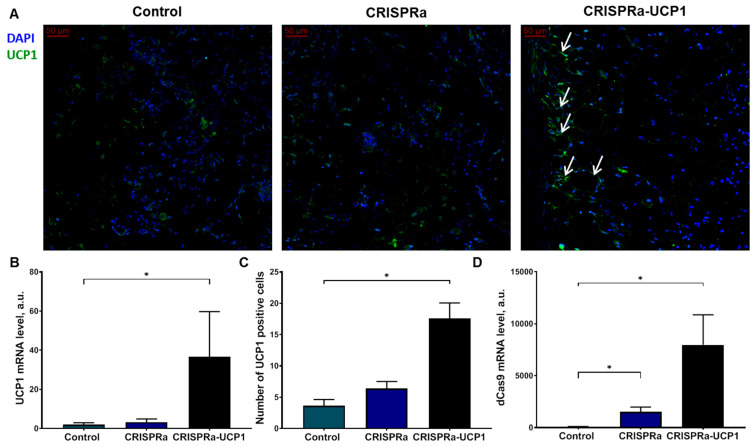
CRISPRa-UCP1 increases the number of UCP1-positive transplanted adipocytes. (**A**) Representative images of Matrigel sections stained for UCP1 and DAPI; (**B**) quantification of UCP1 mRNA in grafts; (**C**) quantification of UCP1-positive adipocytes in graft sections; (**D**) quantification of dCas9 mRNA in grafts. DAPI—4′,6-diamidino-2-phenylindole; UCP1—uncoupling protein 1. Data are represented as mean ± SEM, Kruskel–Wallis test with post-hoc Dunn’s test, *—significance threshold *p* < 0.05.

We found that UCP1 mRNA and the number of UCP1-positive adipocytes were significantly elevated in grafts with adipocytes modified with CRISPRa-UCP1, confirming the efficiency of the CRISPRa system for endogenous UCP1 regulation (Figure 2A–C). As a control for non-specific effects of the system, we transduced adipocytes with baculovirus, containing all CRISPRa components except UCP1-sgRNAa. We did not detect changes in UCP1 expression in these constructs but found that expression of dCas9 remained at a high level 10 days after transduction, suggesting the possibility of a longer duration of CRISPRa effects (Figure 2D). 

### 2.3. CRISPRa-UCP1 System Does Not Alter Morphology or Protein Expression in Transplanted Adipocytes

The transduction of mature adipocytes instead of undifferentiated progenitor cells is beneficial due to the reduction in time from transduction to transplantation, which is important for baculovirus with limited expression time [27]. Moreover, transduction can initiate pro-inflammatory phenotypes or alter differentiation of ADSCs [28]. Adipocytes, unlike ADSCs, instead lose immunoregulatory properties [29]. On the other hand, transplantation of adipocytes is more complicated due to the difficulty of detachment, fragility and floating. To ascertain that mature adipocytes remain functional in grafts we analyzed adipocyte morphology and the expression of hormone-sensitive lipase (HSL), which is usually recruited to the surface of LDs [30], and transcription factor PGC1α, important for white and beige adipogenesis [31]. 

**Figure 3:**
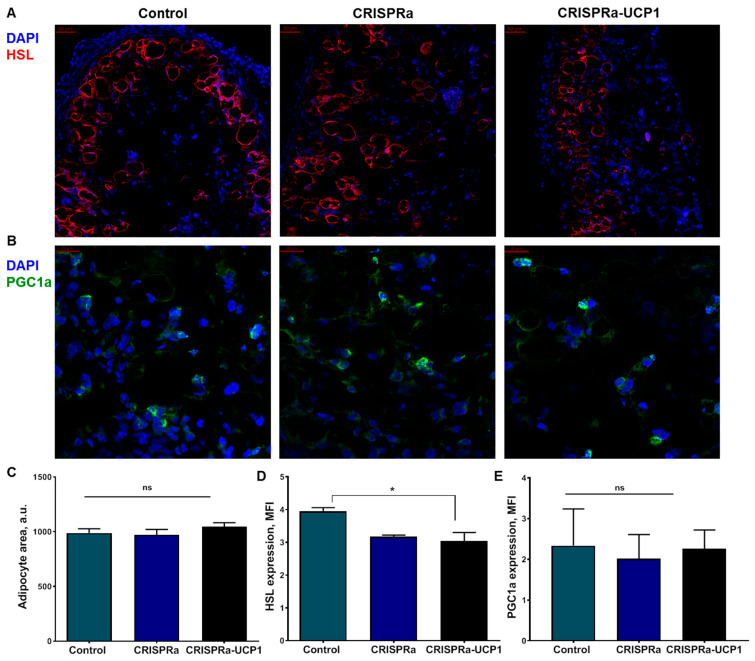
CRISPRa system does not influence adipocyte size or expression of HSL or PGC1α. (**A**) Representative images of graft sections stained for HSL and DAPI, scale bar 50 μm; (**B**) representative images of graft sections stained for PGC1α and DAPI, scale bar 20 μm; (**C**) quantification of average adipocyte area in sections stained for HSL; (**D**) quantification of HSL expression in adipocytes; (**E**) quantification of PGC1α expression in adipocytes. DAPI—4′,6-diamidino-2-phenylindole; HSL—hormone sensitive lipase; MFI—mean fluorescence intensity; PGC1α—peroxisome proliferator-activated receptor gamma coactivator-1 α. Data are represented as mean ± SEM, Kruskel–Wallis test with post-hoc Dunn’s test, *—significance threshold *p* < 0.05. ns—non-significant difference.

Sections of tissue-engineered constructs contained distinctive round empty loci at sites of LD [32] with peripheral HSL staining, which allowed us to calculate their size (Figure 3A). Transduction of CRISPRa and CRISPRa-UCP1 had no influence on adipocyte size (Figure 3C). This contradicts our expectations of a decrease in the number and size of LDs during thermogenesis. Nonetheless, the expression of HSL was slightly decreased in CRISPRa-UCP1, but showed no difference in comparison to CRISPRa (Figure 3D). PGC1α was expressed in cytoplasm and in the nucleus of cells in tissue-engineered constructs and was not affected by CRISPRa or CRISPRa-UCP1 constructs, suggesting no activation of adipogenesis or mitochondrial biogenesis by CRISPRa-UCP1 (Figure 3B,E).

### 2.4. Transplantation of CRISPRa-Engineered Adipocytes Has No Effect on Systemic Glucose Metabolism

According to previous studies, activation of UCP1 expression in adipocytes affects systemic metabolism, decreases fat mass and FBG, and increases glucose tolerance and insulin sensitivity [5,17,18]. To test this, we analyzed the main metabolic characteristics of mice after transplantation of UCP1-expressing adipocytes.

**Figure 4:**
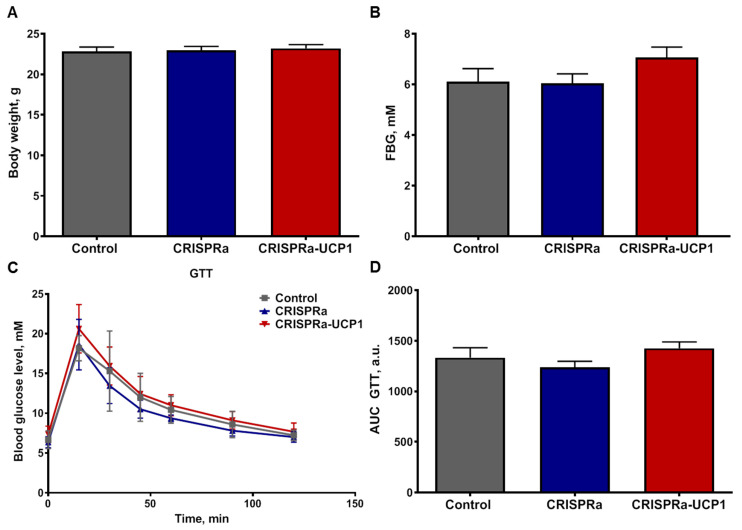
Metabolic parameters of recipient mice 7 days after transplantation of CRISPRa-modified adipocytes. (**A**) Body weight; (**B**) fasting blood glucose level (FBG); (**C**) kinetics of blood glucose level during GTT; (**D**) the area under the curve (AUC) of GTT. GTT—glucose tolerance test. Data are represented as mean ± SEM, Kruskel–Wallis test with post-hoc Dunn’s test, significance threshold *p* < 0.05.

First, we measured basal metabolic parameters in the fasting state—body weight and FBG at 7 days after adipocyte transplantation. We found that CRISPRa- and CRISPRa-UCP1-modified adipocytes had no influence on basal metabolic parameters in mice (Figure 4A,B). Dynamic analysis of systemic response to glucose administration (GTT) also demonstrated equal glucose tolerance in both the control and CRISPRa-UCP1 groups (Figure 4C,D). Thus, we suggest the absence of short-term systemic effects of CRISPRa-UCP1-tissue-engineered constructs on healthy mice.

### 2.5. CRISPRa-UCP1-Modified Tissue Construct Does Not Induce Inflammation and Attracts Host Vasculature

Transplantation of non-autologous cells, transduced with baculovirus and expressing components of the CRISPRa system, can induce inflammation in grafts, potentially turning into systemic inflammation. We analyzed the infiltration of grafts with CD68^+^ macrophages, which are recruited into tissues with active inflammation or cell death. 

**Figure 5:**
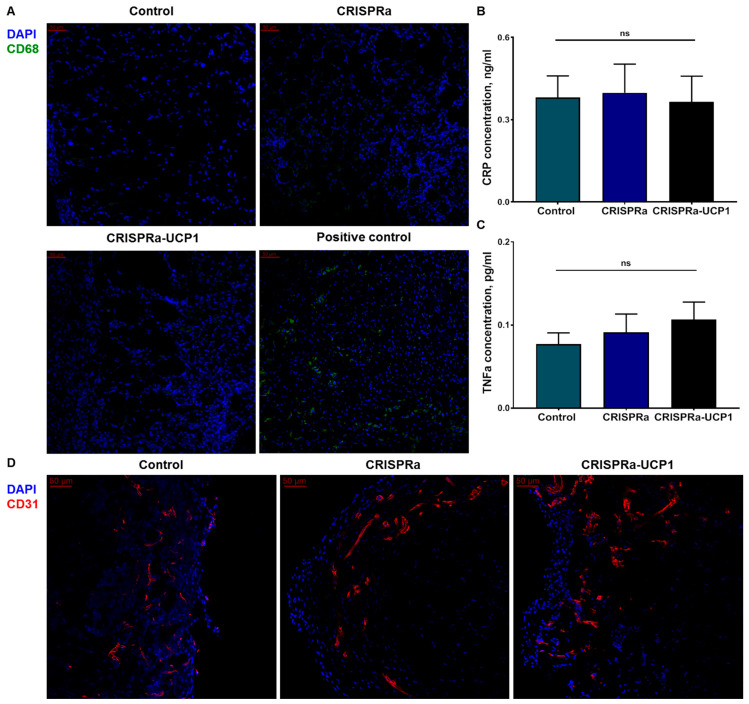
Transplantation of CRISPRa-UCP1-modified adipocytes does not induce inflammation. (**A**) Representative images of graft sections stained for CD68 and DAPI, scale bar 50 μm; (**B**) concentration of C-reactive protein in plasma 8 days after transplantation; (**C**) concentration of tumor necrosis factor a in plasma 8 days after transplantation; (**D**) representative images of graft sections stained for CD31 and DAPI, scale bar 50 um. Data are represented as mean ± SEM, Kruskel–Wallis test with post-hoc Dunn’s test; ns—non-significant difference.

During local inflammation assessment, we did not detect macrophage infiltration of grafts in any groups (Figure 5A). As a positive control for CD68 staining, we analyzed macrophage infiltration of *M. tibialis* after acute ischemia-reperfusion injury, which has previously been demonstrated to increase CD68^+^ cell recruitment. Transplantation of CRISPRa-engineered adipocytes did not increase levels of pro-inflammatory cytokine TNFα or CRP, the marker of acute-phase inflammation in the plasma of animals, confirming the absence of systemic inflammation (Figure 5B,C). Transplantation of Matrigel-based tissue-engineered constructions is usually accompanied by vascularization. In our study, grafts isolated after 8 days of implantation contained CD31^+^ capillary-like structures in the periphery (Figure 5D), reflecting the initiation of vascularization, which is the basis for integration of CRISPRa-UCP1-modified grafts in systemic circulation. 

## 3. Methods

### 3.1. Isolation, Culture and Adipogenic Differentiation of Murine ADSC

Mouse ADSCs were isolated from subcutaneous white adipose tissue dissected from inguinal and anterior depots of euthanized 5-week-old male C57BL/6 mice. The isolation of ADSC was performed as previously described [33]. Briefly, adipose tissue was dissected and digested with collagenase I (200 U/mL, Sigma-Aldrich, St. Louis, MO, USA), following filtration through 100 µm cell strainer for stromal vascular fraction isolation. Cells were seeded and subcultured in standard DMEM high-glucose medium (Gibco, Waltham, MA, USA), supplemented with 10% fetal bovine serum (HyClone, Logan, UH, USA) and PenStrep (100×, Gibco, Waltham, MA, USA). ADSCs of passage 2 were cultured to confluency and exposed to adipogenic inducers (0.5 mM dexamethasone, 0.25 μM isobutylmethylxanthine, 2 μM rosiglitazone and 100 nM insulin, all (Sigma-Aldrich, St. Louis, MO, USA) for 10 days. Cell cultures were tested for mycoplasma contamination by PCR kit MycoReport (#MR001, Evrogen, Moscow, Russia) before transplantation in mice.

### 3.2. Generation of CRISPRa-Engineered Adipocytes

The construction of CRISPRa plasmid—encoding dCas9 and MPH complex (MCP-p65-HSF1 fusion)—and sgRNAa plasmid—encoding human U6 promoter, spacer insertion linker and the sgRNAa scaffold with two copies of MS2 binding aptamers—has been described in detail previously [27]. Spacer sequences for sgRNAa, specific to the region from −400 to −50 relative to the transcription start site of UCP1 gene, were designed using the www.benchling.com online tool (Gilbert, 2014, Benchling, San Francisco, CA, USA). Four 20-nt spacer sequences with the highest targeting efficiency and specificity scores were chosen (Table 1); respective oligonucleotides were synthesized, annealed and inserted into the spacer insertion linker in the sgRNAa plasmid. The four UCP1-targeting sgRNAa were assembled as an sgRNAa array in a single plasmid by BioBrick assembly (restrictases XbaI, NheI, XhoI and Sibenzyme, Novosibirsk, Russia). To prepare the donor plasmid for baculovirus production, the CRISPRa module and assembly of sgRNAa specific to UCP1 were cloned to the baculovirus donor plasmid pFastBacDual (Thermo Fisher Scientific, Waltham, MA, USA). For production of control baculovirus constructs, an encoding CRISPRa module without sgRNAa, was designed.

Donor plasmids were used for recombinant bacmid production in DH10Bac *E. coli* cells (Thermo Fisher Scientific, Waltham, MA, USA) according to the Bac-To-Bac protocol. Next, baculoviruses CRISPRa and CRISPRa-UCP1 were produced in Sf9 insect cells using Cellfectin II Reagent (#10362100, Invitrogen, Waltham, MA, USA) and amplified up to 10^8^ plaque-forming units/mL by infection of Sf9 as previously described [34]. 

For production of adipocytes with increased expression of UCP1, ADSCs were transduced after adipogenic differentiation. Mature adipocytes were incubated with baculovirus particles at multiplicity of infection 50 and shaken on a rocking plate at 28 °C for 4 h, as previously described [29]. The control adipocytes were incubated in Sf9 culture medium Sf-900 III SFM (Gibco, Waltham, MA, USA). After transduction, adipocytes were cultured in the presence of 3 mM sodium butyrate for 24 h and then in standard ADSC culture medium for 18 h before transplantation in mice.

### 3.3. Western Blotting

CRISPRa-modified mature adipocytes were lysed on ice in RIPA buffer (150 mM NaCl, 1% Triton X-100, 0.5% sodium deoxycholate, 0.1% sodium dodecyl sulfate, 50 mM Tris–HCl, pH 8.0) supplemented with a cocktail of protease and phosphatase inhibitors (Roche, Basel, Switzerland). The samples were incubated in Laemmli sample buffer at 95 °C for 5 min for the disruption of possible UCP1 dimeric form. The proteins were separated by Laemmli SDS-PAGE and transferred into a polyvinyliden difluoride membrane under 1 A/h. The membranes were blocked in 5% non-fat dry milk (Applichem, Darmstadt, Germany) on TBST for 4 h and incubated overnight with UCP1 (A5857, Abclonal, Wuhan, China) or vinculin (ab18058, Abcam, Waltham, MA, USA) antibodies. The membranes were stained with HRP-conjugated secondary goat anti-rabbit IgG (ab6721, Abcam, Waltham, MA, USA) or goat anti-mouse IgG (#115-035-146, Jackson ImmunoRes, West Grove, PA, USA), respectively. The protein bands were visualized using the Clarity ECL kit (Bio-Rad, Hercules, CA, USA) and FusionFX gel-documenting system (Vilber Lourmat, Collégien, France) in video mode. Optical density quantification was performed using the GelAnalyzer 19.1 software (www.gelanalyzer.com, accessed on 1 July 2021; software by Istvan Lazar Jr., and Istvan Lazar Sr., CSc; Budapest, Hungary).

### 3.4. Thermogenesis Assessment

For the evaluation of adipocyte thermogenic activity, we used the temperature-sensitive live cell dye ERthermAC (Merck, Darmstadt, Germany) according to the method of Kriszt and coauthors [35]. Modified adipocytes were incubated with 250 nM ERthermAC over 30 min at 37 °C in high-glucose DMEM. Afterwards, incubation cells were washed with PBS and medium was replaced on phenol red-free high glucose DMEM. Visualization was performed on a Leica Stellaris 5 confocal microscope (Leica Microsystems, Wetzlar, Germany) in CO_2_ with a temperature-controlled camera (Okolab,c/o Sviluppo Campania - Lotto 34, 80078 Pozzuoli NA, Italy). ERthermAC fluorescence intensity was measured at 5% CO_2_ and 25 °C, excitation wavelength—555 nm, emission—565–750 nm. Imaging was initiated and 100 μM isoproterenol (ISO; I6504, Sigma) was added after 5 min. Cells were recorded every 15 min over 120 min, using LASX Navigator and Autofocus options. The fluorescence intensity was analyzed using LASX software. Thermogenesis activation in response to ISO was calculated as (F0 − Ft)/F0, where F0 is the basal fluorescence intensity and Ft is the intensity at t min after ISO addition.

### 3.5. Lipid Droplet Size Analysis

For the evaluation of lipid droplet (LD) formation, we used the fluorescent lipophilic dye BODIPY493/503 (Invitrogen, USA). Live cells were stained with 0.25 ug/mL BODIPY493/503 over 20 min. After that, cells were washed with PBS and imaged in phenol red-free DMEM. Imaging was performed using a Leica Stellaris 5 confocal microscope (Leica, Wetzlar, Germany) in CO_2_ and temperature-controlled camera (Okolab, Italy). The LD size and number were analyzed in FIJI software (version 1.53c, Wayne Rasband, NIH, Cambridge, MA, USA) according to the protocol of Exner and co-authors [36].

### 3.6. Animal Housing

Adult male C57BL/6 mice (10 weeks old) were purchased from “Andreevka” animal husbandry facility, Russia. Mice were kept under standard pathogen-free conditions with food and water ad libitum and a regular 12:12 light–dark cycle. Before the initiation of the experiments, animals were matched by body weight and fasting blood glucose (FBG) level (body weight reference range was from 17 to 23 g; FBG reference range was from 3.9 to 6.9 mM) then randomized into 3 groups (7–8 animals per group). For the blood glucose measurements, glucometer Contour Plus One (Ascensia Diabetes Care, Basel, Switzerland) was used. The animal experiment was performed in accordance with the EU Directive 2010/63/EU for animal experiments and was approved by the Institutional Ethics Board for Animal Care (National Medical Research Center for Cardiology, permit #385.06.2009).

### 3.7. CRISPRa-Engineered Adipocyte Transplantation in Animals

Adipocytes after transduction were washed with phosphate buffered saline (PBS) twice and incubated with accutase for 10 min at room temperature. The enzyme was inactivated by addition of warm culture medium and incubation at 37 °C for 5 min. Adipocytes were gently resuspended and centrifuged at 200 g for 10 min. After centrifugation, both sediment and floating cells were collected and resuspended in fresh medium with adipogenic inductors up to 10^7^ cells/mL. Adipocytes were immediately mixed with Corning Matrigel Basement Membrane Matrix (#356237, Corning, Corning, NY, USA) 1:1 on ice and injected (300 µL per mouse) with a 23 G needle into the inguinal fat depot of each recipient mouse under anesthesia (2.5% avertin solution intraperitoneally). After 7 days, mice underwent body weight and FBG measurements and glucose tolerance tests (GTT) as described previously. Briefly, overnight-fasted mice were injected intraperitoneally with sterile 10% glucose solution at 2 g/kg; blood glucose level was measured at 15, 30, 45, 60 and 90 min after glucose administration. On day 8 after adipocytes transplantation, overnight-fasted mice were anesthetized by intraperitoneal injection of 2.5% avertin solution and whole-blood samples were collected from the left ventriculum in 20% EDTA solution with consequent centrifugation (3000 g, +4 °C, 30 min) and supernatant collection. Blood plasma samples were frozen and stored at −80 °C. After that, mice were sacrificed and grafts were collected and frozen in liquid nitrogen.

### 3.8. Immunohistochemistry of CRISPRa-Engineered Adipose Tissue Constructs

Tissues were cryopreserved in Tissue-Tek O.C.T. Compound (Sakura Finetek, Alphen aan den Rijn, The Netherlands), cut into 20 µm sections and used for immunofluorescence staining. For immunofluorescence tissue sections were fixed in 4% paraformaldehyde for 30 min at room temperature and blocked in 10% serum of secondary antibody donor for 1 h at room temperature. The sections were then incubated with primary antibodies overnight at 4°C: UCP1 (1:100, #WH256795, Abclonal, Wuhan, China), HSL (1:100, #4107, Cell Signaling, Danvers, MA, USA), PGC1α (1:200, #ab191838, Abcam, Cambridge, UK), CD68 (1:100; #137001; Biolegend, San Diego, CA, USA) and CD31 (1:100; #50274, BD Biosciences Pharmingen, San Diego, CA, USA). After primary antibody incubation, the sections were washed with PBS and incubated with the appropriate secondary antibody (Alexa Fluor488-conjugated secondary antibody (#A21206); Alexa594-conjugated secondary antibody (#A11032); Thermo Fisher Scientific, Waltham, MA, USA) at a 1:200 dilution for 1 h. After washing with PBS, sections were mounted in Vectashield with DAPI (Vector, ZB0324, Burlingame, CA, USA). Sections were imaged using a Leica Stellaris 5 confocal microscope (Leica, Wetzlar, Germany). The number of UCP1-positive cells was calculated in 5 independent fields of view per animal. HSL and PGC1α expression was evaluated in 5 independent fields of view per animal as mean fluorescence intensity (MFI) in FIJI software.

### 3.9. Gene Expression in CRISPRa-Engineered Adipose Tissue Constructs

Total RNA was extracted and purified from engineered adipose grafts using a spin column kit CleanRNA Standard (#BC033, Evrogen, Moscow, Russia). RNA (500 ng) was treated with DNAse I (#EN0525, Thermo Fisher Scientific, Waltham, MA, USA) and reverse transcribed with RevertAid RT Reverse Transcription Kit (#K1691, Thermo Fisher Scientific, Waltham, MA, USA). Real-time PCR was performed using SYBR green qPCR reagents (#R-414, Syntol, Moscow, Russia) with forward and reverse oligonucleotide primers (400 nM each) in the StepOnePlus Real-Time System (Applied Biosystems, Waltham, MA, USA). Expression of b-actin was used to normalize gene expression. Real-time PCR primer sequences are listed in Table 2.

### 3.10. Enzyme-Linked Immunosorbent Assay (ELISA)

The concentrations of the inflammatory markers C-reactive protein (CRP) and tumor necrosis factor α (TNFα) were measured in mouse plasma samples using ELISA kits for CRP (#SEA821Mu, Cloud-Clone Corp., Wuhan, China) and for TNFα (#MTA00B, R&D Systems, Little Falls, NY, USA). Plasma samples were collected as described above (Section 3.6) and analyzed according to manufacturer’s protocols.

### 3.11. Statistical Analysis

The comparisons among the three groups were carried out using a one-way Kruskel–Wallis test with post-hoc Dunn’s multiple comparison test using GraphPad Prism 8.0. Data are shown as the mean values ± standard error of the mean (SEM); *p* values less than 0.05 were considered statistically significant. AUCs were calculated by Gagnon’s method [37].

## 4. Discussion

Thermogenesis in adipose tissue is a promising therapeutic target to combat obesity, type 2 diabetes and metabolic syndrome. Activation of thermogenic gene expression in adipocytes, following transplantation in vivo, was demonstrated in previous studies using different gene therapy approaches. Here, we develop the CRISPRa transcription regulatory system for activation of UCP1 expression and deliver it in mature adipocytes using a safe and efficient baculovirus vector. We describe in detail adipocyte modification, the state of tissue-engineered constructs after transplantation and their systemic effects on glucose metabolism and inflammation. 

Previously, the CRISPRa system was commonly delivered using plasmids, lentivirus or adeno-associated viruses [17,23,38,39,40]. However, all these methods have limitations: plasmid transfection has low efficiency and duration of expression in primary cells and especially adipocytes, while adeno-associated and lentiviruses have limited insert sizes (4.7 kb and 9 kb, respectively) [41,42]. Researchers normally overcome the limitations of insert capacities by splitting large size transgenes between several vectors, or by packing up to 15 kb inserts into the lentivirus system, which significantly decreases transduction efficiency and virus yield [41,43]. Moreover, lentiviral vectors are not preferred for translational gene and cell therapy due to genome integration, alteration of the cell cycle and activation of immune response [41,44,45].

Due to a large transgene capacity of up to 38 kb [26], the baculovirus expression system was chosen to deliver the CRISPRa system, including dCas9, MPH complex of MCP-p65-HSF1 proteins, and four sgRNAas targeting UCP1 promoters, in one vector. We demonstrated that CRISPRa-UCP1 enhances UCP1 expression in adipocytes and increases thermogenic response to β-adrenergic stimulation with ISO (Figure 1A–D). Furthermore, the expression of UCP1 remains significantly increased and adipose-tissue-engineered constructs contain UCP1-positive adipocytes 8 days after transplantation (Figure 2). 

Expression of UCP1 in mature adipocytes as well as in precursor cells is essential, because UCP1 needs both fatty acids for H^+^ transport and the high activity of metabolism found in adipocytes [46,47]. We investigated the influence of CRISPRa modification on adipocyte morphology and expression of proteins related to adipocyte metabolism. First, we hypothesized that thermogenesis activation could decrease LD accumulation and LD size. In adipocyte cell cultures, CRISPRa-UCP1 decreased the number of medium-sized LDs (Figure 1E,F), but did not induce a prominent shift in LD morphology. These data suggest the absence or low activation of lipolysis. However, thermogenic adipocytes are able to use different energetic substrates to support thermogenesis [48].

Further, we confirmed that tissue-engineered constructs contain mature adipocytes with LDs (Figure 3A). Transplanted adipocytes express the transcription factor PGC1α, regulating white and beige adipogenic differentiation [36], and HSL, detected in the periphery of LDs (Figure 3A,B) [49]. According to previous studies, HSL is activated and recruited into lipid droplets for lipolysis in thermogenic adipocytes [35,50]. Lipolysis provides adipocytes with fatty acids for further oxidation and generation of mitochondrial H^+^ gradients, used by UCP1 for heat production [14]. Furthermore, lipolysis is often supported by LD fragmentation, which increases the substrate availability for lipases [8]. In our study, CRISPRa-induced UCP1 expression did not affect LD area and slightly decreased the expression of HSL in comparison to non-transduced cells (Figure 3A,C,D). CRISPRa-modified cells (without sgRNAa) also had a downward trend in HSL expression in comparison to the control. Probably, the decrease in HSL expression is the result of baculovirus transduction, or interaction with viral particles or expression of CRISPRa components, but not with UCP1 upregulation. Short-term UCP1 expression in adipose-tissue-engineered constructs can be the reason for the absence of the expected effect on markers of lipolysis. 

Activation of thermogenesis in brown and beige adipose tissue leads to significant metabolic changes. Various studies report decreases in body weight and FBG and increases in glucose tolerance under thermogenesis activation [4,5,17]. We examined the parameters of glucose metabolism in lean mice 7 days after transplantation of adipose-tissue-engineered constructs. CRISPRa and CRISPRa-UCP1-modified adipocytes did not change such metabolic parameters as body weight, FBG, AUC or curve shape in GTT (Figure 4). Transplantation of adipocytes transduced with baculovirus and the expression of the CRISPRa system and sgRNAs had no negative effects on systemic metabolism; all metabolic parameters were compliant with target values for healthy lean mice [51]. Our results are inconsistent with studies reporting activation of glucose utilization in animal models with activated UCP1 expression. We suggest that the absence of metabolic effects from UCP1-expressing adipocytes may be the result of an insufficient number of transplanted cells, the short-term exposure or the absence of β-adrenergic stimulation, which activates thermogenesis in vitro. Indeed, in previous studies the effect of UCP1 expression was analyzed at 4–15 weeks after activation or transplantation. Moreover, the most prominent effects were observed in models of obesity and glucose intolerance caused by high-fat feeding [17,18]. In our study, we investigated the short-term effect of UCP1-expressing adipocyte transplantation, due to limitations on transgene expression time. Baculovirus is a non-integrating virus; bacmid degrades over 2–3 weeks inside mammalian cells. The highest expression is often observed for 3–7 days after transduction and decreases to control levels in 14–21 days [28]. The expression can be prolonged by developing the system for site-specific transgene integration in the genome or by producing Cre/loxP hybrid baculovirus [26,28]. Thus, the baculovirus-delivered CRISPRa system does not alter glucose metabolism. An increase in the exposure time of adipose-tissue-engineered constructs may enhance systemic metabolic effects due to the integration of CRISPRa-UCP1-modified grafts into systemic circulation which was initiated in the periphery of grafts on day 8 (Figure 5D).

Since viral vectors, components of the CRISPRa system and non-autologous cell transplantation can be immunogenic, we controlled for local inflammation in the graft and markers of systemic inflammation. The adipose-tissue-engineered constructs were not infiltrated with CD68^+^ cells, confirming the absence of macrophage recruitment (Figure 5A) [52,53]. This confirms that transplantation of cells transduced in vitro can be preferable in comparison to direct viral vector injection, known to activate inflammation [54]. Moreover, the lack of CD68^+^ macrophage infiltration suggests the absence of cell death in the transplanted cells [55]. The markers of systemic inflammation, CRP and TNFα, did not increase in mice after transplantation of unmodified adipocytes or CRISPRa-engineered constructs (Figure 5B,C) and did not exceed the common values reported for healthy mice in other studies [56,57]. Thus, transplantation of CRISPRa-engineered adipocytes does not stimulate inflammation in mice.

## 5. Conclusions

In conclusion, we confirmed that the CRISPRa system is a powerful tool for activation of UCP1 expression in adipocytes. The baculovirus delivery system efficiently transduces adipocytes and provides the expression of the CRISPRa system for at least 10 days. Mature adipocytes in tissue-engineered constructs retain integrity and morphology after transplantation. Tissue-engineered adipocytes with elevated expression of UCP1 had no effect on systemic glucose metabolism due to the short time of in vivo graft exposure and the absence of metabolic abnormalities in the mice. Future development of prolonged expression of UCP1 in adipocytes can improve CRISPRa-UCP1 for successful metabolic regulation in animal models. The absence of local and systemic inflammation in mice after transplantation and the presence of graft vascularization makes the baculovirus-delivered CRISPRa system an attractive gene therapy tool, which can step into clinical application in the future.

## Figures and Tables

**Table 1:** sgRNAa targeting mus musculus UCP1 promoter.

sgRNA	Sequence	Bp Upstream of UCP1 Transcription Start Site
1	TCCACAGCTAGAATAGCTGT	353
2	ACAAAAGGCACCACGCTGCG	252
3	CAGTTCCTGATTATGCCCAG	215
4	AGGGCTTTGGGAGTGACGCG	156

**Table 2:** Real-time PCR primer sequences.

mRNA	Forward Primer	Reverse Primer
b-actin	AGACCTTCAACACCCCAGCCAT	GGATGGCGTGAGGGAGAGCATA
UCP1	ACTGCCACACCTCCAGTCATT	CTTTGCCTCACTCAGGATTGG
dCas9	GGCTACGCCGGATACATTGA	CTCTTGCCGCCTGAGGATAG

## Data Availability

The raw data supporting the conclusions of this article will be made available by the authors, without undue reservation.

## Abbreviations

ADSC	adipose-derived stem cells;
ANOVA	analysis of variance;
AUC	area under curve;
CRISPR	clustered regularly interspaced short palindromic repeats;
CRISPRa	CRISPR activation technology;
CRP	C-reactive protein;
DAPI	4′,6-diamidino-2-phenylindole;
DMEM	Dulbecco’s modified Eagle’s medium;
dCas9	dead CRISPR associated protein 9;
ELISA	enzyme-linked immunosorbent assay;
FBG	fasting blood glucose;
GTT	glucose tolerance test;
HSF1	heat shock factor 1;
HSL	hormone-sensitive lipase;
ISO	isoproterenol;
LD	lipid droplets;
MCP	MS2 coat protein;
MFI	mean fluorescence intensity;
PBS	phosphate-buffered saline;
PGC1α	peroxisome proliferator-activated receptor gamma coactivator-1 α;
SEM	standard error mean;
sgRNA	single guide RNA;
T2DM	type 2 diabetes mellitus;
TAG	triacylglycerides;
TNFα	tumor necrosis factor α;
UCP1	uncoupling protein 1
